# Placental mesenchymal dysplasia: a rare case associated with second trimester fetal growth restriction

**DOI:** 10.1186/s12884-024-06960-8

**Published:** 2024-11-25

**Authors:** Thomas Chadwick, Jennifer Davis, Wafa Bitar, Susmita Pankaja

**Affiliations:** Obstetrics & Gynecology department, Whiston Hospital, Prescot, L35 5DR UK

**Keywords:** Placental mesenchymal dysplasia, Fetal growth restriction, Beckwith-Weidemann syndrome, Molar pregnancy, Placentalomegaly

## Abstract

**Background:**

Placental mesenchymal dysplasia (PMD) is a rare, benign, placental disorder characterised macroscopically by an enlarged multi-cystic placenta. It is a condition associated with a range of reported clinical outcomes and can be misdiagnosed as a molar or partial molar pregnancy given the similarities in clinical presentation. We present an unusual case of PMD complicated by fetal growth restriction and oligohydramnios in the second trimester.

**Case presentation:**

A 33 year old female was referred to our fetal medicine unit with a multi-cystic placenta at dating scan suspected initially to be a partial molar pregnancy. She opted for conservative management and declined invasive testing. At 16 weeks severe fetal growth restriction was present with an estimated fetal weight < 3rd centile and associated with oligohydramnios. Whilst live births in cases of PMD have been reported in the literature, to our knowledge there are no reported successful outcomes in cases of early onset growth restriction at this gestation. The patient opted to proceed with termination of pregnancy given the suspected poor prognosis. Post mortem results confirmed a diagnosis of PMD and fetal growth restriction with normal genetic testing.

**Conclusions:**

Placental mesenchymal dysplasia can be a difficult condition to manage, particularly when counselling about prognosis and deciding whether to continue the pregnancy. More evidence is needed about prognostic factors in PMD associated with a successful outcome. Early onset fetal growth restriction and/or oligohydramnios prior to 20 weeks are likely poor prognostic factors which should be considered in the antenatal counselling.

## Introduction

Placental mesenchymal dysplasia (PMD) is a rare placental disorder associated with stem villous cystic dilation and vesicle formation, leading to distinctive macroscopic and microscopic features [1, 2]. PMD is often misdiagnosed as a molar pregnancy or a partial hydatidiform mole due to its similarities in clinical presentation. However, PMD has different clinical implications and management strategies, with successful pregnancy outcomes being reported [3]. Uncertainty regarding the diagnosis and prognosis in PMD can make the condition difficult to manage [4].

## Case presentation

We present the case of a 33-year-old female referred to our fetal medicine unit (FMU) and ultimately diagnosed with PMD. She was para 1 having had a previous uncomplicated pregnancy with delivery via elective caesarean section at term. Her BMI was 20.7 and she was a nonsmoker. She booked at 10 weeks and 6 days and went on to have a dating scan at 12 weeks and 2 days which showed a single viable intrauterine pregnancy with an enlarged multi-cystic placenta and hence was referred to our FMU for a suspected partial molar pregnancy. Her first trimester combined screen was low chance for trisomies 13, 18 and 21 risk with a HCG of 245.64 U/L (5.08 corrected MoM) and Papp-A 5008.13 mU/l (1.75 corrected MoM).

**Fig. 1:**
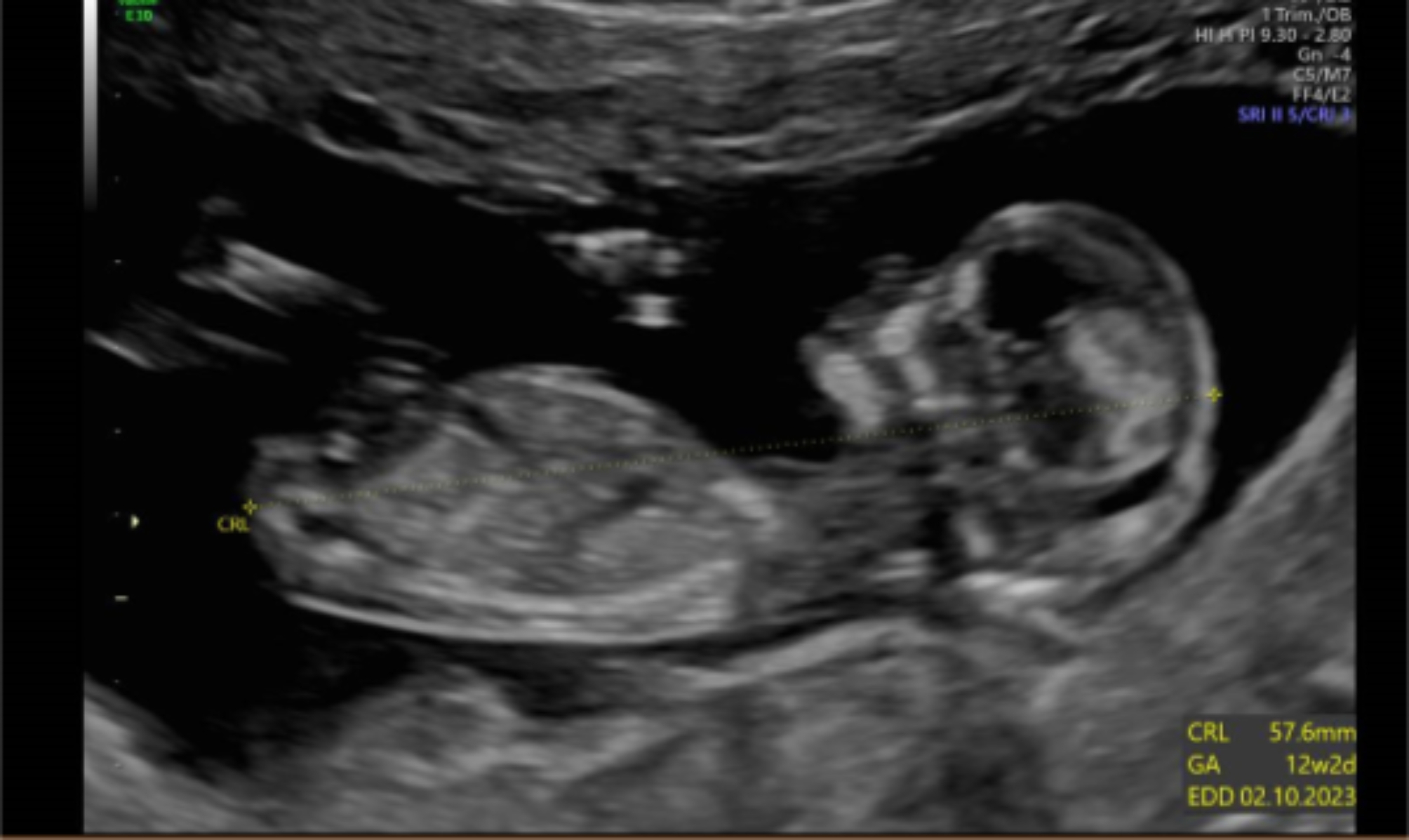
Dating scan at 12 weeks showing a multicystic placenta with an apparently normal fetus

The initial ultrasound scan in FMU confirmed placentalomegaly with a multicystic ‘grape-like’ appearance to the placenta (Fig. 1). The fetal anatomy was unremarkable at this stage allowing for the gestation. The differential diagnosis including partial molar pregnancy and PMD was discussed, and invasive testing offered. The patient opted for conservative management with a rescan in 4 weeks.

**Fig. 2:**
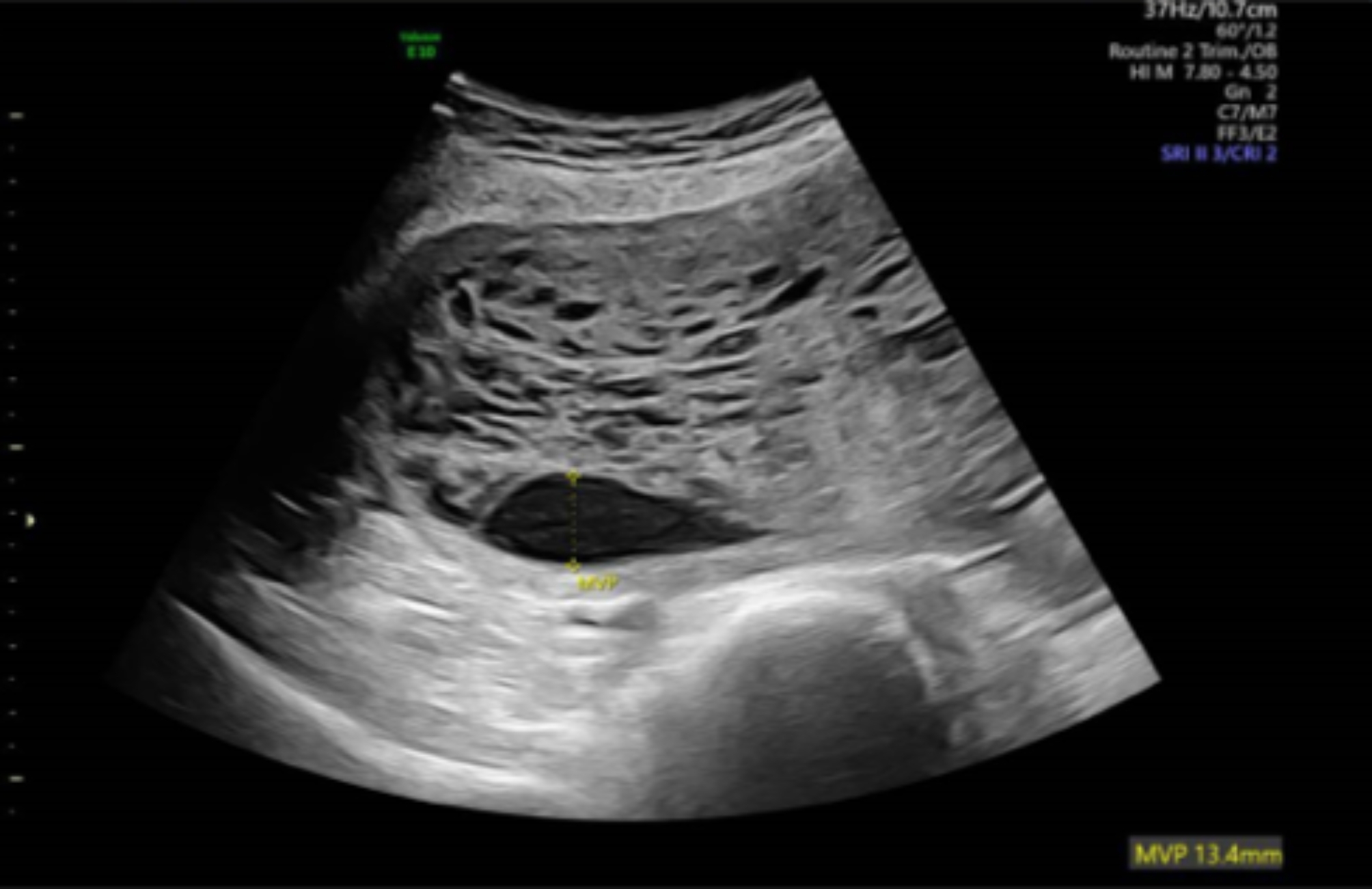
Ultrasound scan at 16 weeks showing an enlarged multicystic placenta and oligohydramnios

Repeat ultrasound at 16 weeks and 1 day again showed a significantly enlarged and multi-cystic placenta (Fig. 2). Fetal biometry using Hadlock showed an estimated fetal weight < 3rd centile. There was oligohydramnios with a maximum pool depth of 13.4 mm. Allowing for the suboptimal views secondary to oligohydramnios the fetal anatomy was unremarkable. Options were again discussed including invasive testing, conservative management or termination of pregnancy with the patient ultimately opting for the latter.

She received medical management with mifepristone and misoprostol and subsequently delivered a female infant with no signs of life. The placenta delivered spontaneously.

**Fig. 3:**
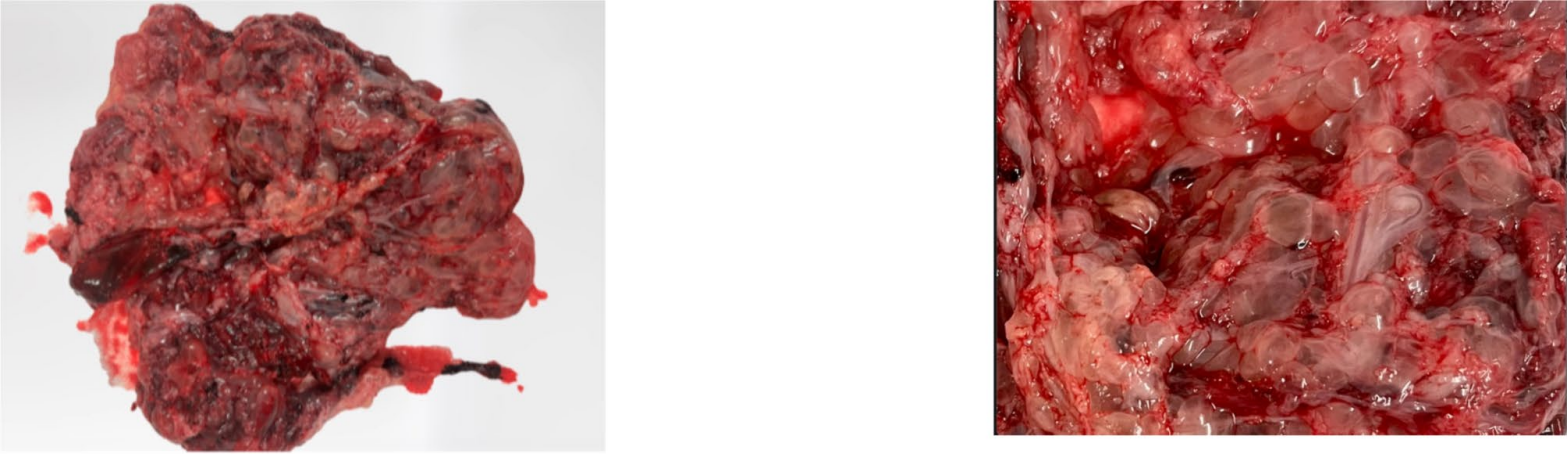
Macroscopic placental examination demonstrating multiple large vesicles on the maternal surface

A full postmortem was performed which showed evidence of fetal growth restriction (fetal weight 48.1 g equivalent to 14 weeks gestation) with abnormalities including mild hypotelorism, low set ears and fixed upper limb deformities. The placenta weighed 181 g (normal range for 16w 77–80 g) with multiple large vesicles noted on gross inspection (Fig. 3). Microscopic examination showed parenchyma comprised of very immature chorionic villi with classical features of PMD including villous enlargement, myxoid stroma, cistern formation within proximal/stem villi and patchy stromal cell karyomegaly, without evidence of trophoblastic hyperplasia. On immunohistochemistry the chorionic villi showed diffuse loss of stromal cell p57 immunoreactivity, consistent with PMD. QFPCR and microarray of the umbilical cord sample showed a normal female genotype with no currently plausible pathological variants being identified. Genetic testing for 11p15 was negative and Beckwith-Weidemann syndrome (BWS) was not confirmed.

## Discussion

PMD is a rare, benign, placental condition with a reported incidence of 0.02% [5]. The condition is often misdiagnosed as a molar pregnancy due to the presence of vesicular changes and placental enlargement.

with the final diagnosis requiring histological examination of the placenta [6]. As highlighted in this case, PMD should be included in the differential diagnosis in all cases where a cystic-appearing placenta is identified.

Differentiating PMD from its mimics can be challenging, with evidence to suggest that the condition is underdiagnosed antenatally [4]. There are however some important features that can help aid diagnosis. Aside from the placental abnormality, PMD is typically associated with a normal fetus, except in cases affected by BWS, approximately 25% of cases, when features such as macrosomia, exomphalos and/or cranio-facial features may be present [5, 6]. Whilst partial molar pregnancies can have a similar placental appearance on ultrasound with grape-like vesicles and placentalomegaly, the cystic changes tend to be less prominent than those seen in PMD or complete molar pregnancies [7]. The fetus in a partial molar pregnancy is typically growth restricted, or the gestational sac can be anembryonic. Differentiation between these conditions can be made on invasive testing with partial hydatidiform moles demonstrating a triploid karyotype, most commonly XXY [2]. HCG levels can also be helpful in such cases with higher levels being typically seen in molar pregnancies, however HCG levels can also be raised in cases of PMD [5]. In our case the HCG was > 5 MoM but below the level that would typically be expected in molar pregnancy. The biomarker alpha-feto protein has also been shown to be raised in cases of PMD, and in paternally derived triploidy, but was not performed in this case [8].

A particular challenge can be found in differentiating PMD from a complete molar pregnancy with a normal co-twin. Both have similar sonographic appearances and will often both be associated with a normal karyotype. Features that may suggest this on ultrasound include the presence of a separating membrane, and/or the presence of a second normal-appearing placenta, however these features can be difficult to identify [2]. Given the associated finding of early onset fetal growth restriction (FGR) in this case, the differential diagnosis, in addition to a partial molar pregnancy and PMD, included a complete molar pregnancy with the co-twin being affected by a discordant pathology associated with FGR. The potential aetiologies for second trimester FGR are broad, including aneuploidies such as trisomy 16, single gene mutations, non-genetic syndromes, and other placental, maternal, or infective causes.

Counselling regarding prognosis in cases of suspected PMD, and the decision regarding conservative management versus termination of pregnancy also presents a challenge, particularly given reported successful outcomes, albeit with a lack of evidence regarding favorable prognostic factors. This contrasts with cases of partial molar pregnancy which are inevitably fatal in the perinatal period [7]. The rates of adverse pregnancy outcomes in PMD are high and include fetal growth restriction, spontaneous preterm delivery, hypertensive disorders, placental abruption, fetal anaemia, intrauterine fetal death and early neonatal death [2, 4]. It is apparent from reviewing existing case reports that growth restriction typically develops after 20 weeks and most commonly presents in the third trimester [4]. The case we present therefore appears particularly rare given that the sequalae of placental dysfunction occurred early in the pregnancy with evidence of fetal growth restriction and oligohydramnios being present at 16 weeks.

With regards to overall perinatal outcome, a 2013 systemic review of pregnancies affected by PMD found that only 9% of cases were uncomplicated with regards to either maternal or neonatal complications [3]. Given the findings of early onset FGR and associated oligohydramnios the prognosis in our case appeared poor which was reflected in the counselling and decision to proceed with termination of pregnancy. We suggest that FGR and oligohydramnios prior to 20 weeks in cases of suspected PMD should be considered poor prognostic factors as to our knowledge there are no reported cases of successful pregnancy outcomes affected by FGR and/or oligohydramnios prior to 20 weeks [4]. Fortunately, the risk of recurrence in subsequent pregnancies appears to be negligible, especially in cases not affected by BWS [8].

## Conclusion

Placental mesenchymal dysplasia can be a difficult condition to manage, particularly when counselling about prognosis and deciding whether to continue the pregnancy. More evidence is needed about prognostic factors in PMD associated with a successful outcome. Early onset FGR and/or oligohydramnios prior to 20 weeks are likely poor prognostic factors which should be considered in the antenatal counselling.

## Data Availability

All data generated or analysed during this study are included in this published article.

## Abbreviations

PMD: Placental mesenchymal dysplasia
BWS: Beckwith-Weidemann syndrome
HCG: Human chorionic gonadotropin
FGR: Fetal growth restriction
